# Remote temporal wavepacket narrowing

**DOI:** 10.1038/s41598-019-39689-y

**Published:** 2019-02-28

**Authors:** Karolina Sedziak-Kacprowicz, Mikołaj Lasota, Piotr Kolenderski

**Affiliations:** Faculty of Physics, Astronomy and Informatics, Nicolaus Copernicus University, Grudziadzka 5, 87-100 Toruń, Poland

## Abstract

Quantum communication protocols can be significantly enhanced by careful preparation of the wavepackets of the utilized photons. Following the theoretical proposal published recently by our group, we experimentally demonstrate the effect of remote temporal wavepacket narrowing of a heralded single photon produced via spontaneous parametric down-conversion. This is done by utilizing a time-resolved measurement on the heralding photon which is frequency-entangled with the heralded photon. We then investigate optimal photon pair source characteristics to minimize heralded wavepacket width.

## Introduction

The phenomenon of entanglement can certainly be seen as the essence of quantum theory. It leads to a plethora of counterintuitive effects, allowing for substantial improvement of numerous applications, particularly in the field of quantum communication^1^. Interestingly, entanglement of different physical properties of light can be utilized in different ways. For example, polarization entanglement is an important resource for protocols of secure information transfer^2–4^, while spatial entanglement is essential for ghost imaging^5,6^ and spatial quantum state encoding^7–9^. Spectral entanglement, on the other hand, can be utilized to improve quantum communication (QC) security^4,10^ or the accuracy of remote clock synchronization^11^.

Photonic implementations of QC protocols suffer from many device imperfections that plague realistic single-photon sources, communication channels and detection systems. Their combination limits the maximal secure distance of information transfer. One of the most effective ways to reduce this limitation is to use temporal filtering to decrease the amount of noise registered by single-photon detectors^12^. Unfortunately, the full potential of this method cannot be used if the single-photon wavepacket is affected by temporal broadening. This turns out to be a common problem in fiber-based QC systems, where the signals propagate through dispersive media.

In order to counter this problem one may try to control the temporal properties of photons emitted by the source. For sources based on the spontaneous parametric down-conversion (SPDC) process, which are particularly useful for quantum communication applications, there are several methods to do so. However, most of them rely on sending at least one of the photons from every SPDC pair through some kind of spectral or temporal modulator, which acts as a filter^13–16^. Therefore, the efficiency of those schemes is significantly reduced, which can be seen as a serious drawback from the perspective of practical quantum communication. Furthermore, some of the methods for temporal shaping of SPDC photons require using specifically designed setup elements^16,17^. For this reason their utilization in broad applications does not seem to be probable in the foreseeable future.

However, a new method to significantly reduce the problem of temporal broadening was proposed recently by our group^10^. It is based on appropriate preparation of spectrally correlated photon pairs^18–23^ and their subsequent time-resolved detection. This method does not require using any highly-specific setup elements. Furthermore, it does not introduce any type of additional filtering to the SPDC photons. This means that the heralding efficiency of temporally-narrowed photons is in principle the same as in the case when our method is not used.

In this work, we experimentally investigate the task of remote preparation of a single-photon wavepacket by a SPDC source. Following the theoretical proposal published in ref.^10^, we show how adjusting the spectral entanglement and applying a time-resolved heralding procedure can substantially narrow the wavepacket of the propagated photons. We also discuss the problem of optimizing the SPDC source for applications utilizing telecommunication fibers of a given length and dispersion coefficient. We discuss our results in the context of improving the performance of quantum key distribution (QKD) schemes.

**Figure 1:**
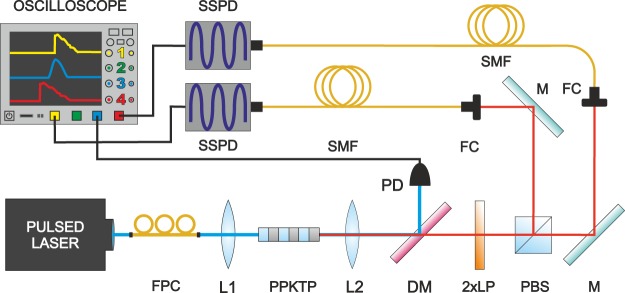
The experimental setup. Pulses from the femtosecond Ti:Sapphire laser are coupled to a fiber. Their polarization is set by a fiber polarization controller FPC. The pump is focused using the lens L1 (plano-convex F = 125 mm) in the type II PPKTP nonlinear crystal. The resulting photon pairs are collimated using the lens L2 (plano-convex, F = 70 mm). A dichroic mirror DM (Semrock FF875-Di01) separates the unconverted laser beam and the SPDC photons. Two longpass filters LP (Semrock BLP01-1319R-25) remove the remainder of the pump. Next, a polarizing beam splitter PBS separates the SPDC photons based on their polarization. They are subsequently coupled to single mode fibers SMF (Corning SMF28e+) using fiber collimators FC (F = 8.0 mm) and mirrors M. The oscilloscope monitors the outputs of superconducting nanowire single-photon detectors SSPD and the fast photodiode PD.

The experimental setup utilized to measure the arrival time distribution of the photon pairs is depicted in Fig. 1. The parametric down conversion process takes place in a type-II 10 mm-long PPKTP crystal with a poling period of 46.2 *μ*m, designed for collinear phase matching 780 nm → 2 × 1560 nm. The crystal is pumped by a pulsed Ti:Sapphire laser coupled to a single-mode fiber, with central wavelength 780.1(1) nm and repetition rate 80.14(2) MHz. A dichroic mirror is used to separate the pump beam and a photon pair. The photodiode illuminated with the pump beam delivers the reference signal, carrying information on the emission time of every pair. Subsequently, the orthogonally-polarized SPDC photons are separated by a polarizing beam splitter and coupled into two 10 km-long standard single-mode fibers (SMFs). The measured value of the group velocity dispersion is $2\beta =-\,2.27\times {10}^{-26}\frac{{{\rm{s}}}^{2}}{{\rm{m}}}$ at 1560 nm, which corresponds to 17.6 ps/(nm ⋅ km). The longpass filters remove the remaining pump beam. The photons are detected using superconducting nanowire detectors (SSPDs). An oscilloscope measures the electric pulses from the SSPDs and the photodiode, and records the respective waveforms, which are post-processed in order to obtain the time stamps for the photon detection. The triggering system is set to record only coincidences from the two SSPD channels in a given time window. The resulting data set is composed of pairs of photon detection times referenced to the laser pulse. The detection system and electronics contribute to the timing jitter, with standard deviation on the order of 45 ps for the SSPDs and around 4 ps for the photodiode.

## Results

### Experimental wavepacket narrowing

**Figure 2:**
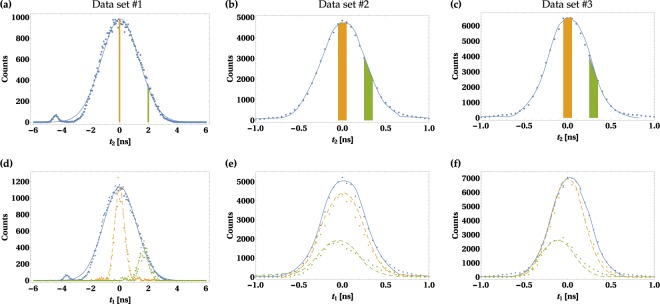
Distribution of arrival times of the two SPDC photons. The top (bottom) row shows the distribution of arrival times of the heralding (heralded) photon. Blue dots (lines) represent experimental data (fitted theoretical model given by Eq. (12)). Each column corresponds to one of the three data sets specified in Table 1. The orange and green colored areas on panels (a)–(c) denote arbitrarily chosen, 100 ps wide detection time windows for the heralding photon. The measured (calculated) distribution of heralded photon arrival times corresponding to those selected areas are plotted on panels (d)–(f) with dots (dashed lines) of the the same colors. The numbers of coincidences in the heralded photons peaks are scaled for convenient presentation of the wavepacket narrowing effect. On panels (d)–(f), the total number of coincidences in peaks of dashed yellow (green) plots are 125 (48), 865 (368) and 1396 (562).

**Figure 3:**
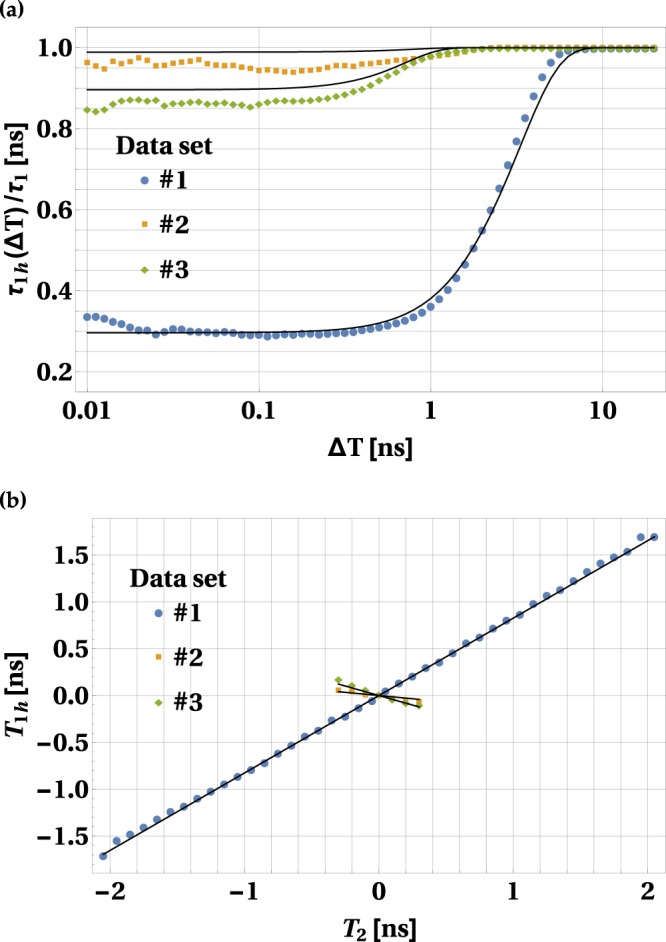
Temporal width and average arrival time of the heralded photon. Panel (a) shows the ratio between the temporal widths of the heralded photon and non-heralded photon, plotted as a function of the width of the detection window for the heralding photon. Panel (b) shows the dependence of the position of the peak of the arrival time distribution function for the heralded photon, *T*_1*h*_, on the position of the peak of the analogous function for the heralding photon, *T*_2_. On both panels the blue, yellow and green dots correspond to the experimental data obtained for the three pump settings specified in the first, second and third column of the Table 1, respectively. The results of theoretical calculation are represented by black solid lines.

**Figure 4:**
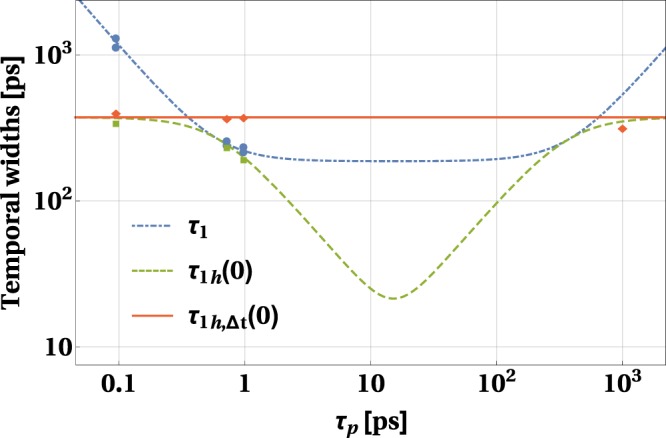
Optimization of SPDC source over the pump laser pulse duration. Temporal widths *τ*_1_ (blue, dot-dashed line), *τ*_1*h*_(0) (green, dashed line) and *τ*_1*h*,Δ*t*_(0) (red, solid line) as functions of *τ*_*p*_, plotted for *σ* = 3.25(1) THz, *L* = 10 km and *β* = −1.15 × 10^−26^ s^2^/m. Blue dots, green squares and red diamonds represent the values of *τ*_1_, *τ*_1*h*_(0) and *τ*_1*h*,Δ*t*_(0) (respectively) obtained in the experiment. The rightmost red diamond corresponds to the experimental value of *τ*_1*h*,Δ*t*_(0) obtained for the CW pump laser.

**Figure 5:**
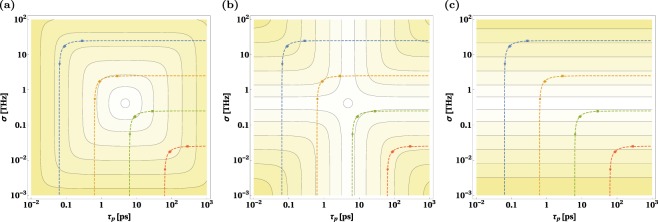
General optimization of SPDC source. Logarithm of the temporal widths (**a**) *τ*_1_, (**b**) *τ*_1*h*_(0) and (**c**) *τ*_1*h*,Δ*t*_(0) plotted as functions of *σ* and *τ*_*p*_. Dashed lines connect pairs of values (*τ*_*p*_, *σ*) corresponding to the following spectral widths of photons emitted from the crystal: *σ*_0_ = 10 GHz (red line), *σ*_0_ = 100 GHz (green line), *σ*_0_ = 1 THz (orange line) and *σ*_0_ = 10 THz (blue line). The colored dots, diamonds and squares denote pairs of points (*τ*_*p*_, *σ*) for which the spectral correlation coefficient takes the value of *ρ* = 0.9, *ρ* = 0 and *ρ* = −0.9 (respectively). The exact connection between the parameters *σ*_0_, *ρ* and *τ*_*p*_, *σ* is explained in the Methods section. The contours shown in each panel have the same value. The lines close to the darkest and lightest filled regions correspond to value −9.2 and −11.3, respectively.

The probability of detecting two SPDC photons, whose spectral wavefunction is given by Eq. (3) in^10^, at time *t*_1_ and *t*_2_ can be described by the following bivariate normal distribution:

1 $$p({t}_{1},{t}_{2})=\frac{1}{2\pi {\tau }_{1}{\tau }_{2}\sqrt{1-{\rho }_{t}^{2}}}\times \exp \,(-\frac{1}{2(1-{\rho }_{t}^{2})}(\frac{{t}_{1}^{2}}{{\tau }_{1}^{2}}+\frac{{t}_{2}^{2}}{{\tau }_{2}^{2}}-\frac{2{t}_{1}{t}_{2}{\rho }_{t}}{{\tau }_{1}{\tau }_{2}})),$$

where *τ*_1_, *τ*_2_ are temporal widths of those photons and *ρ*_*t*_ accounts for temporal correlation between them. In our analysis, we acquire three data sets, consisting of approximately 82 × 10^3^ pairs of photon arrival times, for the three pump settings with different spectral widths, Δ*λ*, specified in Table 1. For each data set, we compute a histogram, to which we subsequently fit the distribution given in Eq. (1). We take into account the background noise in the model, which turned out to be negligible. The parameters are fitted using standard nonlinear model fitting functions. The best fit parameters, *ρ*_*t*_, *τ*_1_ and *τ*_2_ are gathered in Table 1. The measured statistics and fitted functions are depicted with blue dots and solid lines, respectively, in Fig. 2, where the columns correspond to the three data sets specified in Table 1. Note that the error bars are smaller than the markers and the same situation is in Figs 3–5.

**Table 1:** Values of the main parameters.

Data set	#1	#2	#3
Δ*λ*	3.415 (6) nm	0.4491 (82) nm	0.331 (7) nm
*τ*_*p*_	94.58 (16) fs	0.7191 (14) ps	0.976 (2) ps
*ρ*_*t*_	0.9551 (2)	−0.1483 (14)	−0.4443 (11)
*τ*_1_	1.136 (2) ns	0.23607 (24) ns	0.2146 (2) ns
*τ*_2_	1.312 (2) ns	0.25285 (25) ns	0.23130 (21) ns
*τ*_1*h*_/*τ*_1_	29.49 (41)%	96.9 (7)%	87.9 (5)%

The three pump bandwidths, Δ*λ*, utilized in the experiment (standard deviation), the corresponding pulse duration, *τ*_*p*_, the best fit parameters *ρ*_*t*_, *τ*_1_, *τ*_2_ for the statistics of arrival times of SPDC photons to the detectors, and the ratio of reduced temporal width of the heralded photon to the respective temporal width of non-heralded photon, *τ*_1*h*_/*τ*_1_.

Subsequently, we choose two 100 ps-wide detection windows for photon number two (heralding photon) and computed histograms for the measured arrival times of corresponding photon number one (heralded photon). The probability distribution function fitted to the obtained data sets is given by the formula (12) in the Methods section. The results of this fitting procedure are plotted with dashed yellow and green lines on the panels (d)–(f) in Eq. (1). Comparing them with the corresponding blue solid lines we observe that the temporal width of the heralded photon is reduced as compared to the situation when there is no information on the heralding photon arrival time. It implies narrower time distribution of the heralded photon.

The strength of this narrowing effect depends on the temporal correlation between the SPDC photons, which in turn depends on the pump settings. In order to quantify it we introduce the quantity *τ*_1*h*_(Δ*T*), which denotes the temporal width of the heralded photon provided that the detection window for the heralding photon has Δ*T* width. Its theoretical value is simply the standard deviation of the Gaussian function given by formula (12). In general, the higher the absolute value of *ρ*_*t*_, the lower the ratio of *τ*_1*h*_(Δ*T*)/*τ*_1_. This can be seen in Fig. 3(a), where we plot the experimental values of this ratio, obtained through the fitting procedure, as a function of Δ*T*. This data is also compared with the results of the theoretical model depicted with solid lines. The value of *τ*_1*h*_(Δ*T*)/*τ*_1_ is reduced when Δ*T* decreases. However, below a certain threshold value of Δ*T* it approaches a limit, which in theory is given by (see the Methods section)

2 $$\mathop{\mathrm{lim}}\limits_{{\rm{\Delta }}T\to 0}\frac{{\tau }_{1h}({\rm{\Delta }}T)}{{\tau }_{1}}=\sqrt{1-{\rho }_{t}^{2}}.$$

In our experiment, this threshold value can be estimated to be Δ*T* ≈ 300 ps. The strength of the wavepacket narrowing that we were able to observe, calculated in terms of the ratio of *τ*_1*h*_(Δ*T*)/*τ*_1_, is given in Table 1.

The average arrival time of the heralded photon, *T*_1*h*_, depends on the average detection time of the heralding photon, *T*_2_. The experimental results are depicted in Fig. 3(b). In general there is no analytical formula to express this dependence. However, if one chooses a detection window close to the limiting value Δ*T* → 0, the theoretical dependence is linear. It can be derived from Eq. (14), yielding

3 $${T}_{1h}={\rho }_{t}\frac{{\tau }_{1}}{{\tau }_{2}}{T}_{2}$$

To illustrate this, for every *T*_2_ we took fit parameters from Table 1 and numerically calculated *T*_1*h*_. The results of this calculation are depicted by solid black lines in Fig. 3(b).

### Temporal widths of photons as functions of the SPDC source parameters

Now we wish to find the optimal source parameters that could provide us with the narrowest possible wavepackets for a given length of transmission links, *L*, characterized by the dispersion coefficient, *β*. In order to achieve this goal, we first calculate the temporal widths of SPDC photons in terms of the effective phase matching function width^24^, *σ*, and the pump laser pulse duration, *τ*_*p*_, in three different scenarios. The details of these calculations are described in the Methods section. First, we focus on the temporal width of the non-heralded photon, which reads:

4 $${\tau }_{1}=\sqrt{\frac{{\sigma }^{2}{\tau }_{p}^{4}+({\beta }^{2}{L}^{2}{\sigma }^{4}+4){\tau }_{p}^{2}+4{\beta }^{2}{L}^{2}{\sigma }^{2}}{4{\sigma }^{2}{\tau }_{p}^{2}}}.$$

It is not surprising that the value of *τ*_1_ grows to infinity when the pump pulse duration is infinitely short, *τ*_*p*_ → 0, or infinitely long, *τ*_*p*_ → ∞ (CW laser). On the other hand its minimum

5 $${\tau }_{1}^{{\rm{\min }}}=\frac{|\beta |L{\sigma }^{2}+2}{2\sigma },$$

is reached for ${\tau }_{p}^{{\rm{opt}}}=\sqrt{\mathrm{2|}\beta |L}$.

In the asymptotic case, in which the arrival time of the heralding photon is perfectly known, one gets the following expression for the temporal width of the heralded photon:

6 $${\tau }_{1h}\mathrm{(0)}=\sqrt{\frac{({\beta }^{2}{L}^{2}{\sigma }^{4}+4)({\tau }_{p}^{4}+4{\beta }^{2}{L}^{2})}{{\sigma }^{2}{\tau }_{p}^{4}+({\beta }^{2}{L}^{2}{\sigma }^{4}+4){\tau }_{p}^{2}+4{\beta }^{2}{L}^{2}{\sigma }^{2}}}.$$

This quantity also reaches its minimum for ${\tau }_{p}^{{\rm{opt}}}$. It is given by

7 $${\tau }_{1h}^{{\rm{\min }}}\mathrm{(0)}=\frac{2\sqrt{|\beta |L({\beta }^{2}{L}^{2}{\sigma }^{4}+4)}}{|\beta |L{\sigma }^{2}+2}\mathrm{.}$$

On the other hand, for the situation when the emission time of the pump pulse is unknown but the detection time of the heralding photon is available the temporal width of the heralded photon reads:

8 $${\tau }_{1h,\Delta t}\mathrm{(0)=}\frac{\sqrt{{\beta }^{2}{L}^{2}{\sigma }^{4}+4}}{\sigma }\mathrm{.}$$

It is interesting to note that, for a given nonlinear crystal with fixed *σ* parameter, this does not depend on the pump settings.

### Optimizing an SPDC source over the pump pulse duration

Let us first analyze the scenario where the crystal parameter, *σ*, is fixed and the experimenter can modify the pulse duration. The relation between *τ*_1_, *τ*_1*h*_(0) and *τ*_1*h*,Δ*t*_(0) can be clearly seen in Fig. 4, where the theoretically calculated and experimentally measured values of these functions are plotted. It can be seen that when the additional information about the photon pair generation time is available, the respective temporal width is small compared to the case when there is no such information, ${\tau }_{1h}\mathrm{(0)} < {\tau }_{1h,{\rm{\Delta }}t}\mathrm{(0)}$. This can be seen by comparing the solid red and the dashed green curves in Fig. 4.

Furthermore, one can observe that the temporal width of the non-heralded wavepacket is never smaller than the heralded one, *τ*_1*h*_(0) ≤ *τ*_1_. The strength of the narrowing effect, *τ*_1*h*_(0)/*τ*_1_, asymptotically goes to zero for *τ*_*p*_ → 0 and *τ*_*p*_ → ∞. On the other hand, for *τ*_*p*_ = 2/*σ* and *τ*_*p*_ = |*β*|*Lσ* it reaches the maximal value of one. The first maximum can be attributed to the crystal producing spectrally decorrelated photon pairs. The second one is the consequence of the propagation in the fiber. As shown in ref.^10^, the temporal correlation changes with the propagation distance. Therefore the second maximum corresponds to the point where there is no temporal correlation. The local minimum of *τ*_1*h*_(0)/*τ*_1_ is reached for ${\tau }_{p}^{{\rm{opt}}}$. It can be calculated using the formulas (5) and (7). For the case illustrated in Fig. 4, this central minimum within our experimental setting takes the value of approximately 11.3%, meaning that the lowest value of *τ*_1*h*_(0)/*τ*_1_ measured in our experiment, 29.49% (see Table 1), could be further reduced by setting the pulse duration to *τ*_*p*_ = 15.2 ps.

In connection with our previous work^10^, it is interesting to ask what type of spectral correlation we have for the optimal pump settings, for which both *τ*_1_ and *τ*_1*h*_(0) reach their minima. Through simple mathematical calculations, one can derive the following formula for the optimal value of spectral correlation coefficient:

9 $${\rho }^{opt}=\frac{2-|\beta |L{\sigma }^{2}}{2+|\beta |L{\sigma }^{2}}.$$

This implies that for sufficiently short propagation distance the optimal SPDC photons are always positively correlated, while for very long distance communication schemes negative correlation is best. However, the distance at which the type of optimal spectral correlation changes from positive to negative depends on the parameter *σ*.

### Designing the optimal SPDC source

It is also important to know the optimal photon pair source design when one can arbitrarily choose both the crystal and the pump laser settings, *τ*_*p*_ and *σ*, for a given pair of symmetric transmission links characterized by the parameters *L* and *β*. The dependence of the temporal widths *τ*_1_, *τ*_1*h*_(0) and *τ*_1*h*,Δ*t*_(0) on the pump laser settings, plotted for fixed values of *L* and *β*, can be seen in Fig. 5. A simple calculation shows that both *τ*_1_ and *τ*_1*h*_(0) reach their absolute minima for ${\tau }_{p}^{{\rm{opt}}}=\sqrt{\mathrm{2|}\beta |L}$ and ${\sigma }^{{\rm{opt}}}=\sqrt{\mathrm{2/|}\beta |L}$. Those minima are identical and read:

10 $${\tau }_{1}^{{\rm{abs}}}={\tau }_{1h}^{{\rm{abs}}}=\sqrt{\mathrm{2|}\beta |L}.$$

This means that for an optimal SPDC source, the narrowing effect is not present. Therefore, spectrally decorrelated pairs are optimal for quantum communication in this case. For our 10 km-long SMF fiber links, we get ${\tau }_{p}^{{\rm{opt}}}\approx 15.2\,{\rm{ps}}$ and *σ*^opt^ ≈ 132 GHz. Alternatively the absolute minimal value of *τ*_1*h*,Δ*t*_(0) equals

11 $${\tau }_{1h,{\rm{\Delta }}t}^{{\rm{abs}}}(0)=2\sqrt{|\beta |L}.$$

Again, it can be reached for ${\sigma }^{{\rm{opt}}}=\sqrt{\mathrm{2/|}\beta |L}$, but for arbitrary *τ*_*p*_.

## Discussion

The possibility for narrowing temporal widths of photons by performing time-resolved measurements and optimizing the settings of the photon pair source, discussed above, could have multiple applications. As an example, one can consider the problem of long-distance fiber-based QKD. While the first experimental realizations of QKD protocols with pairs of photons created in SPDC process were presented almost 20 years ago^25–27^, very few QKD implementations utilizing long-distance telecommunication fibers and SPDC sources have been demonstrated to date. Furthermore, according to our knowledge, the parameters of the source were not optimized in any way for these implementations. For example, ref.^28^ used pairs of photons created utilizing a CW laser while ref.^29^ used relatively long pump pulses of 1.4 ns temporal width. The detection gates for SPDC photons that were defined in the aforementioned works (2.5 ns-long and 2 ns-long, respectively) were much longer than they could have been if the photon-pair sources were properly optimized. Such optimization would then lead to much higher signal-to-noise ratios and enable generation of secure keys for considerably longer distances separating the participants of the protocol.

In fact, all of the record-breaking long-distance fiber-based QKD implementations reported in the literature in recent times^33–36^ have been realized with weak coherent pulses (WCP) and decoy-pulse method^30–32^, which allows the trusted parties to neutralize multiphoton events. However, there are many papers suggesting that using a heralded single-photon source can be more beneficial for decoy-based QKD, both in the standard^37,38^ and in the measurement-device-independent^39–42^ regimes. One of the limiting factors of WCP sources in this context is that even in the ideal decoy case the amount of truly single-photon pulses that can be obtained from a WCP source cannot be larger than approximately 37% of all the emitted signals. Meanwhile, the heralded single-photon sources based on SPDC process can provide the participants of a QKD protocol with pulses that have much better photon statistics and heralding efficiency exceeding 80%^43–46^. Moreover, by optimizing the settings and performing temporal filtering in the way we demonstrated in our work, one can reduce the temporal width of the emitted photons to a level comparable to the temporal width of WCP photons, without substantially decreasing the efficiency of the source. Contrary to WCP sources, photon-pair sources can also be placed in the middle of the distance separating the trusted parties, forming a symmetric QKD setup configuration, analogous to the scheme analyzed in this paper. The superiority of such a scheme over the standard, highly asymmetric setup configuration in terms of the maximal security distance between the participants of a QKD protocol was theoretically demonstrated in ref.^47^. Taking all of the above considerations into account, one can expect SPDC sources to provide very attractive alternative to WCP for long-distance fiber-based QKD, if optimized properly.

In summary, we investigated the problem of wavepacket shaping of a single photon heralded by the time-resolved detection of the other photon from an SPDC pair. We showed how the strength of the wavepacket narrowing depends on the parameters of the photon pair source and the width of the detection window for the heralding photon. Our theoretical predictions^10^ were compared with the experimental results, showing very good agreement. We experimentally observed the reduction of the width of the heralded wavepacket to approximately 29% as compared to the non-heralding scenario. It should be stressed here that the detection windows used in our experiment were established only for the purpose of showing the wavepacket narrowing. In general case, using free-running detectors with good time-resolving capability, one can detect all of the heralding photons and subsequently perform temporal narrowing of the heralded photons as a postprocessing procedure. In this way it is possible to increase signal-to-noise ratio of the measurement results without introducing any filtering of SPDC photons, which always lead to lower heralding efficiency. Moreover, in this work we performed optimization over the pump laser pulse duration in order to find the minimal possible wavepacket temporal width. For the case of our experimental setup, further narrowing to 11.4 % is feasible for *τ*_*p*_ = 15.2 ps. Finally, we derived formulas for optimal parameters of the SPDC source, minimizing the aforementioned temporal widths. In our work we focused on the scenario, in which the SPDC photons propagate through a pair of identical single-mode fibers. A generalization to the case of fibers with non-equal lengths or different dispersion characteristics is currently under consideration. Further extension of this framework, which would allow for the optimization of quantum communication protocols relying on interference between two photons, *e.g*. quantum teleportation^48,49^ or entanglement swapping^50,51^, is also possible.

## Methods

### Heralded photon’s arrival time in realistic situations

In practice, the detection window for the heralding photon always has finite width. Let us assume that the heralding photon was detected at the time *T*_2_ with uncertainty Δ*T*, which is the width of detection window. In this case, the probability distribution for the arrival time of the heralded photon can be calculated from (1) by utilizing the following expression:

12 $$\begin{array}{rcl}{p}_{c}({t}_{1};{T}_{2},{\rm{\Delta }}T) & = & \frac{{\int }_{{T}_{2}-{\rm{\Delta }}T\mathrm{/2}}^{{T}_{2}+{\rm{\Delta }}T\mathrm{/2}}\,{\rm{d}}{t}_{2}p({t}_{1},{t}_{2})}{\int {\rm{d}}{t}_{1}\,{\int }_{{T}_{2}-{\rm{\Delta }}T\mathrm{/2}}^{{T}_{2}+{\rm{\Delta }}T\mathrm{/2}}\,{\rm{d}}{t}_{2}\,p({t}_{1},{t}_{2})}\\  & = & \frac{{e}^{-\frac{{t}_{1}^{2}}{2{\tau }_{1}^{2}}}({\rm{erf}}\,(\frac{{\tau }_{1}({\rm{\Delta }}T-{T}_{2})+{\rho }_{t}{\tau }_{2}{t}_{1}}{\sqrt{2-2{\rho }_{t}^{2}}{\tau }_{1}{\tau }_{2}})+{\rm{erf}}\,(\frac{{\tau }_{1}({\rm{\Delta }}T+{T}_{2})-{\rho }_{t}{\tau }_{2}{t}_{1}}{\sqrt{2-2{\rho }_{t}^{2}}{\tau }_{1}{\tau }_{2}}))}{\sqrt{2\pi }{\tau }_{1}({\rm{erf}}(\frac{{T}_{2}+{\rm{\Delta }}T}{\sqrt{2}{\tau }_{2}})-{\rm{erf}}(\frac{{T}_{2}-{\rm{\Delta }}T}{\sqrt{2}{\tau }_{2}}))}\mathrm{.}\end{array}$$

Note that Δ*T* → ∞ when the information on the arrival time of the heralding photon is not available to the experimenter. In this case:

13 $$\mathop{\mathrm{lim}}\limits_{{\rm{\Delta }}T\to \infty }\,{p}_{c}({t}_{1};{T}_{2},{\rm{\Delta }}T)\propto \exp \,(-\frac{{t}_{1}^{2}}{2{\tau }_{1}^{2}}).$$

On the other hand, for very short detection window we get:

14 $$\mathop{\mathrm{lim}}\limits_{{\rm{\Delta }}T\to 0}\,{p}_{c}({t}_{1};{T}_{2},{\rm{\Delta }}T)\propto \exp \,(-\frac{{({T}_{2}{\rho }_{t}{\tau }_{1}-{t}_{1}{\tau }_{2})}^{2}}{2(1-{\rho }_{t}^{2}){\tau }_{1}^{2}{\tau }_{2}^{2}}).$$

Therefore, the temporal width of the heralded photon in the asymptotic case of Δ*T* → 0 reads

15 $$\mathop{\mathrm{lim}}\limits_{{\rm{\Delta }}T\to 0}\,{\tau }_{1h}({\rm{\Delta }}T)={\tau }_{1}\sqrt{1-{\rho }_{t}^{2}}.$$

From this expression, we can easily obtain Eq. (2). Furthermore, Eq. (14) allows us to calculate the average detection time of the heralding photon in the case of Δ*T* → 0, which is given by the formula (3).

### Calculation of the temporal widths of SPDC photons

In order to express the temporal widths of SPDC photons in terms of the source parameters *σ* and *τ*_*p*_, we start by assuming a simplified biphoton wavefunction in the following form^23,52^:

16 $$\varphi ({\nu }_{1},{\nu }_{2})=M\,\exp \,(-\frac{{({\nu }_{1}-{\nu }_{2})}^{2}}{{\sigma }^{2}}-\frac{{({\nu }_{1}+{\nu }_{2})}^{2}{\tau }_{p}^{2}}{4}),$$

This expression is similar to the formula (3) in ref.^10^, where we utilized parameters directly describing the properties of the biphoton state, namely its spectral correlation coefficient *ρ* and spectral widths *σ*_1_, *σ*_2_. Those two formulas are equivalent to each other if the crystal produces pairs of photons with equal spectral widths, *i.e. σ*_1_ = *σ*_2_ ≡ *σ*_0_. In this case the comparison between them gives the following transformation:

17 $$\begin{array}{rcl}{\tau }_{p} & = & \frac{1}{{\sigma }_{0}\sqrt{1+\rho }},\\ \sigma  & = & 2{\sigma }_{0}\sqrt{1-\rho }.\end{array}$$

This allows us to rewrite the expressions (8), (10) and (12) for the temporal widths of the heralded and non-heralded wavepackets derived in^10^ in terms of the parameters describing the photon pair source, *σ* and *τ*_*p*_, and transmission links, *β* and *L*. In this way we obtain the formulas (4), (6) and (8).

## References

[1] Yuan Z-S (2010). Entangled photons and quantum communication. Phys. Rep..

[2] Ekert AK (1991). Quantum cryptography based on Bell theorem. Phys. Rev. Lett..

[3] Gisin N, Ribordy G, Tittel W, Zbinden H (2002). Quantum cryptography. Rev. Mod. Phys..

[4] Gisin N, Thew RH (2002). Quantum communication. Nat. Photon..

[5] Pittman T, Shih Y, Strekalov D, Sergienko A (1995). Optical imaging by means of two-photon quantum entanglement. Phys. Rev. A.

[6] Shih, Y. The physics of ghost imaging. Preprint at, https://arxiv.org/abs/0805.1166 (2008).

[7] Solís-Prosser MA, Neves L (2011). Remote state preparation of spatial qubits. Phys. Rev. A.

[8] Solís-Prosser MA (2013). Preparing arbitrary pure states of spatial qudits with a single phase-only spatial light modulator. Opt. Lett..

[9] Varga JJM (2014). Optimized generation of spatial qudits by using a pure phase spatial light modulator. J. Phys. B: At., Mol. Opt. Phys..

[10] Sedziak K, Lasota M, Kolenderski P (2017). Reducing detection noise of a photon pair in a dispersive medium by controlling its spectral entanglement. Optica.

[11] Giovannetti V, Lloyd S, Maccone L (2001). Quantumenhanced positioning and clock synchronization. Nature.

[12] Patel KA (2012). Coexistence of high-bit-rate quantum key distribution and data on optical fiber. Phys. Rev. X.

[13] Bellini M (2003). Nonlocal Pulse Shaping with Entangled Photon Pairs. Phys. Rev. Lett..

[14] Pe’er A, Dayan B, Friesem AA, Silberberg Y (2005). Temporal Shaping of Entangled Photons. Phys. Rev. Lett..

[15] Averchenko V (2017). Temporal shaping of single photons enabled by entanglement. Phys. Rev. A.

[16] Karpiński M, Jachura M, Wright LJ, Smith BJ (2017). Bandwidth manipulation of quantum light by an electrooptic time lens. Nat. Photonics.

[17] Ansari V (2018). Heralded generation of high-purity ultrashort single photons in programmable temporal shapes. Opt. Express.

[18] Kim Y-H, Grice WP (2002). Generation of pulsed polarization entangled two-photon state via temporal and spectral engineering. J. Mod. Opt..

[19] Giovannetti V, Maccone L, Shapiro JH, Wong FNC (2002). Generating Entangled Two-Photon States with Coincident Frequencies. Phys. Rev. Lett..

[20] Kim Y-H, Grice WP (2005). Quantum interference with distinguishable photons through indistinguishable pathways. J. Opt. Soc. Am. B.

[21] Hendrych M, Mičuda M, Torres JP (2007). Tunable control of the frequency correlations of entangled photons. Opt. Lett..

[22] Lutz T, Kolenderski P, Jennewein T (2013). Toward a downconversion source of positively spectrally correlated and decorrelated telecom photon pairs. Opt. Lett..

[23] Lutz T, Kolenderski P, Jennewein T (2014). Demonstration of spectral correlation control in a source of polarization entangled photon pairs at telecom wavelength. Opt. Lett..

[24] Kolenderski P, Wasilewski W, Banaszek K (2009). Modeling and optimization of photon pair sources based on spontaneous parametric down-conversion. Phys. Rev. A.

[25] Jennewein T, Simon C, Weihs G, Weinfurter H, Zeilinger A (2000). Quantum Cryptography with Entangled Photons. Phys. Rev. Lett..

[26] Naik DS, Peterson CG, White AG, Berglund AJ, Kwiat PG (2000). Entangled State Quantum Cryptography: Eavesdropping on the Ekert Protocol. Phys. Rev. Lett..

[27] Tittel W, Brendel J, Zbinden H, Gisin N (2000). Quantum Cryptography Using Entangled Photons in Energy-Time Bell States. Phys. Rev. Lett..

[28] Wang Q (2008). Experimental Decoy-State Quantum Key Distribution with a Sub-Poissionian Heralded Single-Photon Source. Phys. Rev. Lett..

[29] Sun Q-C (2014). Experimental passive decoy-state quantum key distribution. Laser Phys. Lett..

[30] Hwang W-Y (2003). Quantum Key Distribution with High Loss: Toward Global Secure Communication. Phys. Rev. Lett..

[31] Wang X-B (2005). Beating the Photon-Number-Splitting Attack in Practical Quantum Cryptography. Phys. Rev. Lett..

[32] Lo H-K, Ma X, Chen K (2005). Decoy State Quantum Key Distribution. Phys. Rev. Lett..

[33] Liu Y (2010). Decoystate quantum key distribution with polarized photons over 200 km. Opt. Express.

[34] Korzh B (2015). Provably secure and practical quantum key distribution over 307 km of optical fibre. Nat. Photonics.

[35] Yin H-L (2016). Measurement-Device-Independent Quantum Key Distribution Over a 404 km Optical Fiber. Phys. Rev. Lett..

[36] Boaron, A. *et al*. Secure quantum key distribution over 421 km of optical fiber. arXiv:1807.03222 (2018). 10.1103/PhysRevLett.121.190502 30468607

[37] Wang Q, Wang X-B, Guo G-C (2007). Practical decoystate method in quantum key distribution with a heralded single-photon source. Phys. Rev. A.

[38] Zhu J-R, Li J, Zhang C-M, Wang Q (2017). Parameter optimization in biased decoy-state quantum key distribution with both source errors and statistical uctuations. Quantum Inf. Process..

[39] Wang Q, Wang X-B (2013). Efficient implementation of the decoy-state measurement-device-independent quantum key distribution with heralded single-photon sources. Phys. Rev. A.

[40] Zhou Y-Y, Zhou X-J, Su B-B (2016). A measurementdevice- independent quantum key distribution protocol with a heralded single photon source. Optoelectron. Lett..

[41] Zhou X-Y, Zhang C-H, Zhang C-M, Wang Q (2017). Obtaining better performance in the measurementdevice- independent quantum key distribution with heralded single-photon sources. Phys. Rev. A.

[42] Zhang C-H, Zhang C-M, Guo G-C, Wang Q (2018). Biased three-intensity decoy-state scheme on the measurement-device-independent quantum key distribution using heralded single-photon sources. Opt. Express.

[43] Pomarico E, Sanguinetti B, Guerreiro T, Thew R, Zbinden H (2012). MHz rate and efficient synchronous heralding of single photons at telecom wavelengths. Opt. Ex-press.

[44] Pereira MDC (2013). Demonstrating highly symmetric single-mode, single-photon heralding efficiency in spontaneous parametric downconversion. Opt. Lett..

[45] Ramelow S (2013). Highly efficient heralding of entangled single photons. Opt. Express.

[46] Kaneda F, Garay-Palmett K, U’Ren AB, Kwiat PG (2016). Heralded single-photon source utilizing highly nondegenerate, spectrally factorable spontaneous parametric downconversion. Opt. Express.

[47] Ma X, Fung C-HF, Lo H-K (2007). Quantum key distribution with entangled photon sources. Phys. Rev. A.

[48] Bennett CH (1993). Teleporting an Unknown Quantum State via Dual Classical and Einstein-Podolsky-Rosen Channels. Phys. Rev. Lett..

[49] Bouwmeester D (1997). Experimental quantum teleportation. Nature (London).

[50] Żukowski M, Zeilinger A, Horne MA, Ekert AK (1993). “Event-Ready-Detectors” Bell Experiment via Entanglement Swapping. Phys. Rev. Lett..

[51] Pan J-W, Bouwmeester D, Weinfurter H, Zeilinger A (1998). Experimental Entanglement Swapping: Entangling Photons That Never Interacted. Phys. Rev. Lett..

[52] Gajewski A, Kolenderski P (2016). Spectral correlation control in down-converted photon pairs. Phys. Rev. A.

